# 8-Bromo-3,4-dihydro-2*H*-1,3-thia­zino[2,3:2′,1′]imidazo[5′,4′-*b*]pyridine

**DOI:** 10.1107/S1600536810012778

**Published:** 2010-04-14

**Authors:** Hend Bel Ghacham, Youssef Kandri Rodi, Frédéric Capet, El Mokhtar Essassi, Seik Weng Ng

**Affiliations:** a Laboratoire de Chimie Organique Appliquée, Faculté des Sciences et Techniques, Université Sidi Mohamed Ben Abdallah, Fés, Morocco; b Unité de Catalyse et de Chimie du Solide (UCCS), UMR 8181, Ecole Nationale Supérieure de Chimie de Lille, Lille, France; c Laboratoire de Chimie Organique Hétérocyclique, Pôle de Compétences Pharmacochimie, Université Mohammed V-Agdal, BP 1014 Avenue Ibn Batout, Rabat, Morocco; d Department of Chemistry, University of Malaya, 50603 Kuala Lumpur, Malaysia

## Abstract

The imidazopyridine ring system in the title compound, C_9_H_8_BrN_3_S, is almost planar [r.m.s. deviation of the C and N atoms = 0.007 (1) Å]. The S and methyl­ene C atoms connected to the five-membered ring lie within this plane. The remaining two methyl­ene groups of the thia­zine ring are disordered over two sets of sites in a 0.817 (5):0.183 (5) ratio.

## Related literature

The parent triclyclic condensed imidazole (without bromine) has been patented as a pharmaceutical; see: Hideg *et al.* (1975 ▶, 1976 ▶). For other compounds synthesized from 6-bromo-1*H*-imidazo[4,5-*b*]pyridine-2(3*H*)-thione, see: Liszkiewicz *et al.* (2007 ▶); Prasad *et al.* (1986 ▶); Yutilov & Svertilova (1988 ▶).
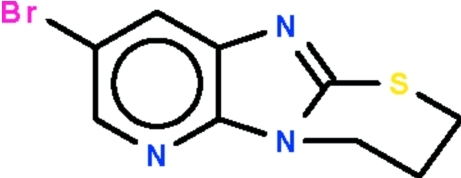


## Experimental

### 

#### Crystal data

C_9_H_8_BrN_3_S
*M* *_r_* = 270.15
Monoclinic, 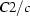

*a* = 20.2738 (3) Å
*b* = 13.2786 (2) Å
*c* = 7.3169 (1) Å
β = 102.193 (1)°
*V* = 1925.33 (5) Å^3^
*Z* = 8
Mo *K*α radiation
μ = 4.45 mm^−1^
*T* = 100 K
0.46 × 0.14 × 0.12 mm

#### Data collection

Bruker X8 APEXII diffractometer
Absorption correction: multi-scan (*SADABS*; Sheldrick, 1996 ▶) *T*_min_ = 0.234, *T*_max_ = 0.618
30460 measured reflections
3561 independent reflections
3003 reflections with *I* > 2σ(*I*)
*R*_int_ = 0.039
Standard reflections: 0

#### Refinement

*R*[*F* ^2^ > 2σ(*F* ^2^)] = 0.024
*wR*(*F* ^2^) = 0.064
*S* = 0.99
3561 reflections
146 parameters
14 restraints
H-atom parameters constrained
Δρ_max_ = 0.49 e Å^−3^
Δρ_min_ = −0.55 e Å^−3^

### 

Data collection: *APEX2* (Bruker, 2008 ▶); cell refinement: *SAINT* (Bruker, 2008 ▶); data reduction: *SAINT*; program(s) used to solve structure: *SHELXS97* (Sheldrick, 2008 ▶); program(s) used to refine structure: *SHELXL97* (Sheldrick, 2008 ▶); molecular graphics: *X-SEED* (Barbour, 2001 ▶); software used to prepare material for publication: *publCIF* (Westrip, 2010 ▶).

## Supplementary Material

Crystal structure: contains datablocks global, I. DOI: 10.1107/S1600536810012778/bt5242sup1.cif

Structure factors: contains datablocks I. DOI: 10.1107/S1600536810012778/bt5242Isup2.hkl

Additional supplementary materials:  crystallographic information; 3D view; checkCIF report

## Figures and Tables

**Fig. 1.**
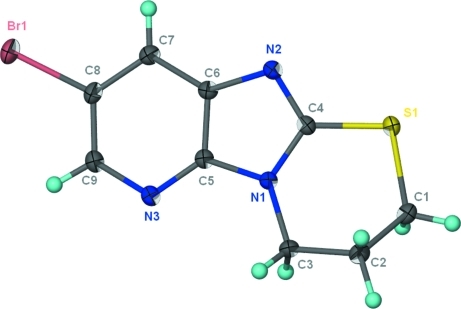
Anisotropic displacement ellipsoid plot (Barbour, 2001) of C9H8BrN3O at the 70% probability level; H atoms are drawn as spheres of an arbitrary radius. The disorder is not shown.

## supplementary crystallographic information

### Comment

Commerically-available 6-bromo-1*H*-imidazo[4,5-*b*]pyridine-2(3*H*)-thione has been used to react with a range of organic compounds to furnish chemicals having useful biological activities (Liszkiewicz *et al.*, 2007; Prasad *et al.*, 1986; Yutilov & Svertilova, 1988). The compound reacts with 1-chloropropanal under catalytic conditions to yield the title triclyclic condensed imidazole (Scheme I, Fig. 1). The imidazopyridine fused ring is planar. One ethylene fragment of the six-membered ring is twisted such that one atom lies above and the other below the plane. This fragment is disordered over two positions.

### Experimental

6-Bromo-1*H*-imidazo[4,5-*b*]pyridine-2(3*H*)-thione (1 mmol), potassium carbonate (4 mmol), tetra-*n*-butylammonium bromide (0.1 mmol) and 1-chloro-propanol (1.5 mmol) in DMF (15 ml) were stirred for 72 h. After completion of reaction (as monitored by TLC), the salt was filtered and the solvent removed under reduced pressure. The resulting residue was purified by column chromatography on silica gel using chloroform/hexane (1/1) as eluent. Colorless crystals were isolated when the solvent was allowed to evaporate.

### Refinement

H atoms were placed in calculated positions (C—H = 0.95–0.99 Å) and were included in the refinement in the riding model approximation, with *U*(H) set to 1.2*U*(C).

The two methylene atoms next to the S atom are disordered over two sites; the disorder refined to an 0.817 (5):0.183 (5) ratio. The pair of S—C distances were restrained to be equal within 0.01 Å of each other, as were the pair of C—C distances. The anisotropic temperature factors of the primed atoms were restrained to be nearly isotropic.

### Crystal data

**Table:**

C_9_H_8_BrN_3_S	*F*(000) = 1072
*M**_r_* = 270.15	*D*_x_ = 1.864 Mg m^−^^3^
Monoclinic, *C*2/*c*	Mo *K*α radiation, λ = 0.71073 Å
Hall symbol: -C 2yc	Cell parameters from 9070 reflections
*a* = 20.2738 (3) Å	θ = 2.8–32.5°
*b* = 13.2786 (2) Å	µ = 4.45 mm^−^^1^
*c* = 7.3169 (1) Å	*T* = 100 K
β = 102.193 (1)°	Block, colourless
*V* = 1925.33 (5) Å^3^	0.46 × 0.14 × 0.12 mm
*Z* = 8	

### Data collection

**Table:**

Bruker X8 APEXII diffractometer	3561 independent reflections
Radiation source: fine-focus sealed tube	3003 reflections with *I* > 2σ(*I*)
graphite	*R*_int_ = 0.039
φ and ω scans	θ_max_ = 32.8°, θ_min_ = 1.9°
Absorption correction: multi-scan (*SADABS*; Sheldrick, 1996)	*h* = −30→30
*T*_min_ = 0.234, *T*_max_ = 0.618	*k* = −20→20
30460 measured reflections	*l* = −11→11

### Refinement

**Table:**

Refinement on *F*^2^	Primary atom site location: structure-invariant direct methods
Least-squares matrix: full	Secondary atom site location: difference Fourier map
*R*[*F*^2^ > 2σ(*F*^2^)] = 0.024	Hydrogen site location: inferred from neighbouring sites
*wR*(*F*^2^) = 0.064	H-atom parameters constrained
*S* = 0.99	*w* = 1/[σ^2^(*F*_o_^2^) + (0.0351*P*)^2^ + 1.6536*P*] where *P* = (*F*_o_^2^ + 2*F*_c_^2^)/3
3561 reflections	(Δ/σ)_max_ = 0.001
146 parameters	Δρ_max_ = 0.49 e Å^−^^3^
14 restraints	Δρ_min_ = −0.55 e Å^−^^3^

### Fractional atomic coordinates and isotropic or equivalent isotropic displacement parameters (Å^2^)

**Table:**

	*x*	*y*	*z*	*U*_iso_*/*U*_eq_	Occ. (<1)
Br1	0.446434 (7)	0.655625 (11)	0.45346 (2)	0.02338 (5)	
S1	0.082325 (17)	0.50817 (3)	0.60465 (5)	0.01612 (7)	
N1	0.20317 (6)	0.43515 (8)	0.54957 (17)	0.0139 (2)	
N2	0.19469 (6)	0.60483 (9)	0.56459 (18)	0.0162 (2)	
N3	0.31734 (6)	0.42166 (9)	0.50005 (18)	0.0167 (2)	
C1	0.07654 (9)	0.37320 (13)	0.6425 (4)	0.0175 (4)	0.817 (5)
H1A	0.0993	0.3568	0.7726	0.021*	0.817 (5)
H1B	0.0285	0.3537	0.6256	0.021*	0.817 (5)
C2	0.10897 (9)	0.31333 (13)	0.5076 (3)	0.0178 (4)	0.817 (5)
H2A	0.0888	0.3342	0.3780	0.021*	0.817 (5)
H2B	0.0991	0.2409	0.5196	0.021*	0.817 (5)
C1'	0.0642 (4)	0.3746 (5)	0.5409 (16)	0.0186 (18)	0.183 (5)
H1'A	0.0233	0.3527	0.5836	0.022*	0.183 (5)
H1'B	0.0556	0.3670	0.4033	0.022*	0.183 (5)
C2'	0.1237 (3)	0.3089 (6)	0.6309 (11)	0.0158 (18)	0.183 (5)
H2'A	0.1106	0.2371	0.6148	0.019*	0.183 (5)
H2'B	0.1360	0.3232	0.7667	0.019*	0.183 (5)
C3	0.18508 (7)	0.32823 (10)	0.5435 (2)	0.0179 (3)	
H3A	0.2062	0.2962	0.6639	0.021*	0.817 (5)
H3B	0.2030	0.2947	0.4433	0.021*	0.817 (5)
H3C	0.2240	0.2887	0.6118	0.021*	0.183 (5)
H3D	0.1748	0.3051	0.4119	0.021*	0.183 (5)
C4	0.16459 (7)	0.51691 (10)	0.57248 (19)	0.0142 (2)	
C5	0.26416 (7)	0.47390 (10)	0.52582 (19)	0.0140 (2)	
C6	0.25763 (7)	0.57918 (10)	0.53377 (19)	0.0143 (2)	
C7	0.31223 (7)	0.63821 (10)	0.5118 (2)	0.0166 (2)	
H7	0.3111	0.7097	0.5143	0.020*	
C8	0.36844 (7)	0.58455 (10)	0.4857 (2)	0.0158 (2)	
C9	0.37001 (7)	0.47932 (11)	0.4812 (2)	0.0175 (3)	
H9	0.4101	0.4471	0.4641	0.021*	

### Atomic displacement parameters (Å^2^)

**Table:**

	*U*^11^	*U*^22^	*U*^33^	*U*^12^	*U*^13^	*U*^23^
Br1	0.01399 (7)	0.02031 (8)	0.03740 (10)	−0.00406 (5)	0.00897 (6)	0.00137 (6)
S1	0.01303 (14)	0.01364 (14)	0.02374 (16)	0.00071 (11)	0.00854 (12)	−0.00036 (12)
N1	0.0120 (5)	0.0103 (4)	0.0206 (5)	−0.0002 (4)	0.0058 (4)	−0.0006 (4)
N2	0.0136 (5)	0.0117 (5)	0.0246 (6)	0.0011 (4)	0.0068 (4)	0.0003 (4)
N3	0.0131 (5)	0.0136 (5)	0.0244 (6)	0.0008 (4)	0.0065 (4)	−0.0004 (4)
C1	0.0156 (8)	0.0145 (7)	0.0240 (11)	−0.0020 (6)	0.0079 (7)	0.0010 (7)
C2	0.0155 (7)	0.0133 (7)	0.0255 (11)	−0.0024 (6)	0.0066 (7)	−0.0022 (6)
C1'	0.020 (4)	0.015 (3)	0.023 (5)	−0.005 (3)	0.009 (3)	−0.001 (3)
C2'	0.016 (3)	0.013 (3)	0.020 (4)	−0.001 (2)	0.005 (3)	0.002 (3)
C3	0.0153 (6)	0.0099 (5)	0.0301 (7)	−0.0009 (4)	0.0088 (5)	−0.0011 (5)
C4	0.0128 (5)	0.0125 (5)	0.0183 (6)	0.0014 (4)	0.0054 (4)	−0.0002 (5)
C5	0.0123 (5)	0.0122 (5)	0.0179 (6)	−0.0001 (4)	0.0043 (4)	−0.0003 (4)
C6	0.0132 (5)	0.0114 (5)	0.0187 (6)	0.0005 (4)	0.0041 (5)	0.0006 (4)
C7	0.0144 (6)	0.0128 (5)	0.0230 (6)	−0.0008 (4)	0.0047 (5)	0.0008 (5)
C8	0.0119 (5)	0.0153 (6)	0.0208 (6)	−0.0022 (4)	0.0046 (5)	0.0011 (5)
C9	0.0128 (6)	0.0160 (6)	0.0250 (7)	0.0010 (5)	0.0067 (5)	0.0002 (5)

### Geometric parameters (Å, °)

**Table:**

Br1—C8	1.8986 (13)	C1'—C2'	1.521 (13)
S1—C4	1.7367 (14)	C1'—H1'A	0.9900
S1—C1	1.8211 (18)	C1'—H1'B	0.9900
S1—C1'	1.851 (6)	C2'—C3	1.536 (6)
N1—C4	1.3689 (17)	C2'—H2'A	0.9900
N1—C5	1.3836 (17)	C2'—H2'B	0.9900
N1—C3	1.4647 (17)	C3—H3A	0.9900
N2—C4	1.3243 (17)	C3—H3B	0.9900
N2—C6	1.3844 (17)	C3—H3C	0.9900
N3—C5	1.3286 (17)	C3—H3D	0.9900
N3—C9	1.3445 (18)	C5—C6	1.4067 (18)
C1—C2	1.521 (3)	C6—C7	1.3931 (19)
C1—H1A	0.9900	C7—C8	1.391 (2)
C1—H1B	0.9900	C7—H7	0.9500
C2—C3	1.522 (2)	C8—C9	1.3982 (19)
C2—H2A	0.9900	C9—H9	0.9500
C2—H2B	0.9900		
			
C4—S1—C1	100.42 (7)	N1—C3—C2	111.68 (12)
C4—S1—C1'	100.1 (3)	N1—C3—C2'	111.7 (3)
C4—N1—C5	105.63 (11)	N1—C3—H3A	109.3
C4—N1—C3	128.79 (12)	C2—C3—H3A	109.3
C5—N1—C3	125.55 (11)	N1—C3—H3B	109.3
C4—N2—C6	103.85 (11)	C2—C3—H3B	109.3
C5—N3—C9	113.77 (12)	H3A—C3—H3B	107.9
C2—C1—S1	111.42 (14)	N1—C3—H3C	109.3
C2—C1—H1A	109.3	C2'—C3—H3C	109.3
S1—C1—H1A	109.3	N1—C3—H3D	109.3
C2—C1—H1B	109.3	C2'—C3—H3D	109.3
S1—C1—H1B	109.3	H3C—C3—H3D	107.9
H1A—C1—H1B	108.0	N2—C4—N1	114.41 (12)
C1—C2—C3	112.43 (15)	N2—C4—S1	121.96 (10)
C1—C2—H2A	109.1	N1—C4—S1	123.61 (10)
C3—C2—H2A	109.1	N3—C5—N1	126.67 (12)
C1—C2—H2B	109.1	N3—C5—C6	127.66 (13)
C3—C2—H2B	109.1	N1—C5—C6	105.67 (11)
H2A—C2—H2B	107.8	N2—C6—C7	131.50 (12)
C2'—C1'—S1	110.1 (6)	N2—C6—C5	110.44 (12)
C2'—C1'—H1'A	109.6	C7—C6—C5	118.06 (12)
S1—C1'—H1'A	109.6	C8—C7—C6	114.94 (12)
C2'—C1'—H1'B	109.6	C8—C7—H7	122.5
S1—C1'—H1'B	109.6	C6—C7—H7	122.5
H1'A—C1'—H1'B	108.2	C7—C8—C9	122.59 (13)
C1'—C2'—C3	111.2 (6)	C7—C8—Br1	119.37 (10)
C1'—C2'—H2'A	109.4	C9—C8—Br1	118.04 (10)
C3—C2'—H2'A	109.4	N3—C9—C8	122.97 (13)
C1'—C2'—H2'B	109.4	N3—C9—H9	118.5
C3—C2'—H2'B	109.4	C8—C9—H9	118.5
H2'A—C2'—H2'B	108.0		
			
C4—S1—C1—C2	−42.19 (16)	C1—S1—C4—N1	10.57 (15)
C1'—S1—C1—C2	49.0 (7)	C1'—S1—C4—N1	−12.8 (4)
S1—C1—C2—C3	67.0 (2)	C9—N3—C5—N1	−179.92 (13)
C4—S1—C1'—C2'	44.5 (7)	C9—N3—C5—C6	0.4 (2)
C1—S1—C1'—C2'	−48.5 (7)	C4—N1—C5—N3	179.51 (14)
S1—C1'—C2'—C3	−69.0 (8)	C3—N1—C5—N3	−2.5 (2)
C4—N1—C3—C2	17.2 (2)	C4—N1—C5—C6	−0.78 (15)
C5—N1—C3—C2	−160.32 (14)	C3—N1—C5—C6	177.23 (13)
C4—N1—C3—C2'	−19.3 (4)	C4—N2—C6—C7	179.69 (15)
C5—N1—C3—C2'	163.2 (3)	C4—N2—C6—C5	−0.67 (16)
C1—C2—C3—N1	−52.1 (2)	N3—C5—C6—N2	−179.37 (14)
C1—C2—C3—C2'	44.8 (5)	N1—C5—C6—N2	0.92 (16)
C1'—C2'—C3—N1	54.3 (7)	N3—C5—C6—C7	0.3 (2)
C1'—C2'—C3—C2	−42.7 (5)	N1—C5—C6—C7	−179.37 (12)
C6—N2—C4—N1	0.15 (16)	N2—C6—C7—C8	178.98 (14)
C6—N2—C4—S1	−178.61 (10)	C5—C6—C7—C8	−0.6 (2)
C5—N1—C4—N2	0.41 (16)	C6—C7—C8—C9	0.3 (2)
C3—N1—C4—N2	−177.51 (14)	C6—C7—C8—Br1	179.90 (10)
C5—N1—C4—S1	179.16 (10)	C5—N3—C9—C8	−0.9 (2)
C3—N1—C4—S1	1.2 (2)	C7—C8—C9—N3	0.6 (2)
C1—S1—C4—N2	−170.77 (14)	Br1—C8—C9—N3	−179.09 (11)
C1'—S1—C4—N2	165.9 (4)		
